# A Challenging Differential Diagnosis Between Brain Radionecrosis and Recurrent Metastatic Disease, with Temporary Clinical/Radiological Response to Bevacizumab and Later Imaging Suspicious for Oligoprogression

**DOI:** 10.3390/life16040552

**Published:** 2026-03-27

**Authors:** Ana Maria Rata, Gabriela Rahnea-Nita, Roxana-Andreea Rahnea-Nita, Mihaela Emilia Dumitru, Alexandru Nechifor, Iulia Chiscop, Dan-Andrei Mitrea, Dorel Firescu, Raluca Barzu, Laura-Florentina Rebegea

**Affiliations:** 1 The Clinical Department, The Faculty of Medicine and Pharmacy, “Dunarea de Jos” University, 800008 Galati, Romania; anamariailie2418@gmail.com (A.M.R.); alexandru.nechifor@ugal.ro (A.N.); iulia.chiscop@ugal.ro (I.C.); dorel.firescu@ugal.ro (D.F.); laura.rebegea@ugal.ro (L.-F.R.); 2 Specific Disciplines Department, The Faculty of Nursing and Midwifery, “Carol Davila” University of Medicine and Pharmacy, 020021 Bucharest, Romania; gabriela.rahnea-nita@umfcd.ro; 3 M Hospital, 010903 Bucharest, Romania; 4 The Clinical Department, The Faculty of Medicine, “Carol Davila” University of Medicine and Pharmacy, 020021 Bucharest, Romania; 5 Radiotherapy Department, “Sfantul Apostol Andrei” Emergency Hospital Galati, 800578 Galati, Romania; 6 NEUROAXIS, 040215 Bucharest, Romania; dan.mitrea@neuroaxis.ro; 7 Live Wins Association, 030923 Bucharest, Romania

**Keywords:** brain radiation necrosis, magnetic resonance perfusion, tumor progression, bevacizumab, long-term survival

## Abstract

Background: Brain radiation necrosis is a side effect of radiotherapy that can occur months, or even years, after the end of treatment. From an anatomical–pathological perspective, it is characterized by avascular damage, demyelination, and necrosis. Methods: We present a case of a patient with breast cancer cT2N1M0 and multiple brain metastases occurring at 2 years after diagnosis, who was treated with whole-brain radiotherapy (WBRT) and Stereotactic Radiotherapy (SRT) for tumor progression. Dynamic imaging revealed right parietal post-therapeutic changes in aggravation, requiring differential diagnosis between tumor progression (TP) and brain radionecrosis (BRN). Results: Brain radionecrosis and tumor progression are difficult to differentiate due to their similar radiological and clinical characteristics. MRI perfusion plays an important role in differentiating the two entities. Conclusions: Differentiating radiation necrosis from a recurrent tumor is crucial for appropriate treatment. Medical management includes corticosteroids as first-line treatment, after which bevacizumab is administered as secondary therapy.

## 1. Introduction

Brain metastases (BMs) are the most common intracranial neoplasm in adults with cancer. It is estimated that 20–40% of patients with tumor malignancies will develop brain metastases during the evolution of diseases [1,2,3,4,5,6].

In breast cancer patients, the risk of intracranial dissemination varies by subtype, depending on hormone receptor status and HER 2 status, with an increased risk in patients with HER2-positive or triple-negative breast cancer [1,7]. Overall survival in cancer patients has increased due to the development of systemic therapies, but this has led to an increase in the incidence of BMs.

Imaging is an essential component in the diagnosis and management of brain metastases, with magnetic resonance imaging (MRI) preferred [1,8]. In recent years, consensus groups have proposed different requirements for brain tumor imaging protocols to diagnose gliomas, brain metastases, and primary central nervous system lymphomas in multicenter studies [1,9].

Regarding follow-up imaging regimens, patients with brain metastases require MRI monitoring every 3–4 months for the first 2 years after initial treatment. If new signs, symptom worsening, or disease progression occur, then MRI should be performed even earlier. Two years after the initial treatment, MRI is performed regularly, and patients with active disease or necrosis require very close imaging follow-up long-term [9].

Radiotherapy provides excellent local control for brain metastases. However, risk of experiencing long-term adverse effects associated with stereotactic radiotherapy (SRT) has increased [1,10,11,12,13]. Advanced imaging techniques, such as magnetic resonance spectroscopy (MRS) or dynamic susceptibility contrast (DSC) or dynamic contrast-enhanced (DCE) perfusion MRI, and amino acid positron emission tomography after stereotactic radiation therapy, can highlight the adverse effects of radiation therapy, which require differential diagnosis with tumor progression (TP) [1,14,15,16,17,18].

Brain radiation necrosis (BRN) is a late complication of treatment. It can occur months, even years after the end of treatment. BRN may be asymptomatic, being detected at follow-up, or may have progressive symptoms that cause significant morbidity [19,20,21].

In this article, we present the case of a patient with breast cancer T2N1M0 Luminal B, treated with neoadjuvant chemotherapy (CMT), conservative surgery, and hormonal treatment (HT), alongside multiple brain metastases occurring after 2 years, treated with whole-brain radiotherapy (WBRT) and SRT. Dynamic imaging revealed right parietal post-therapeutic changes in progression, requiring differential diagnosis between TP and RN.

WBRT treats the entire brain over multiple sessions, covering microscopic disease. SRT delivers high-dose, precise radiation only to tumors in 2–5 sessions, sparing healthy tissue and preserving cognitive function. SRT is preferred for limited metastases, while WBRT handles extensive disease.

## 2. Case Presentation

A 34-year-old patient was diagnosed with left breast cancer in 2018, stage cT2N1M0, Luminal B. She underwent neoadjuvant chemotherapy treatment, surgery-conservative treatment, left sectorectomy, and sentinel lymph node histopathology. The histopathologic result was as follows: ypT0N1(1+/4N) L0V0R0; IHC: RE95%, RP25%; HER2 = 1+; ki67 = 45%; HT. After surgery, the histopathological exam identified lymph node metastases and HER 2 status was negative.

In 2020, the patient presented with multiple brain metastasis and received whole-brain adjuvant radiotherapy (WBRT) in 30 Gy/10 fractions; the systemic treatment administered was a CDK4/6 inhibitor plus Fulvestrant and ovarian suppression therapy.

**Figure 1:**
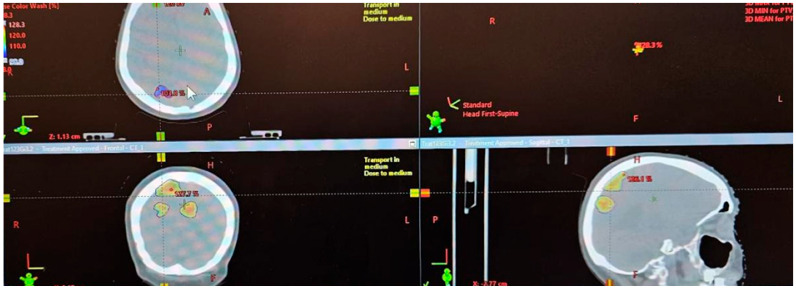
The images are from radiotherapy treatment, in SRT technique, performed in November 2024, 25 Gy/5 fractions for progressive right parietal disease.

In February 2023, the patient received SRT for right parietal and left parietal brain metastasis progression in 21 Gy/3 fractions. In October 2024, cerebral IRM revealed dimensional progression about 18/13 mm of chronic hematic lesions and, consequently, in November 2024, the patient received SRT in 25 Gy/5 fractions for progressive right parietal disease (Figure 1). Dynamic imaging performed at 3 and 6 months after completion of SRT evidenced parietal post-therapeutic changes in aggravation.

The cumulative biological effective dose (BED) for right parietal iradiation was 170 Gy. The median cumulative BED ranges between 120 and 200 Gy assuming an alfa/beta report of 3 Gy for late-reacting normal brain tissue and for symptomatic brain radionecrosis.

**Figure 2:**
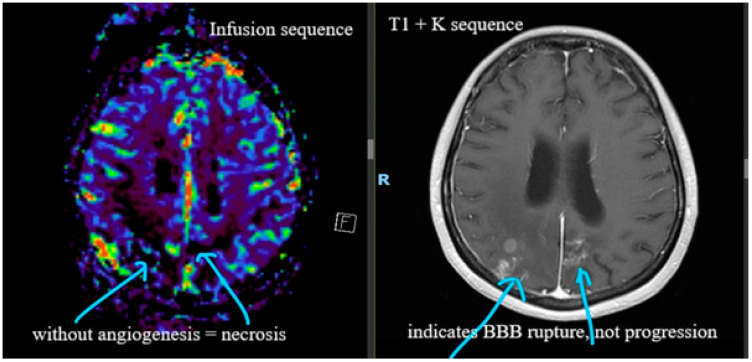
May 2025. Brain perfusion MRI indicating blood–brain barrier (BBB) rupture and no progression.

In May 2025, the patient presented with neurologic hemiparesis classified as clinical status of 4/5 brachial and 3/5 crural; she was able to walk with unilateral support and showed a Karnofsky Performance Status of 60–70%. At that time, the patient sought therapeutic opinions from several centers. She received a final diagnosis of BRN (Figure 2) and a recommendation for off-label treatment with bevacizumab at 5 mg/kg. The decision was made to stop CDK4/6 inhibitor treatment and continue with Fulvestrant and ovarian suppression therapy. Between May and July 2025, she received four cycles of bevacizumab at 400 mg.

**Figure 3:**
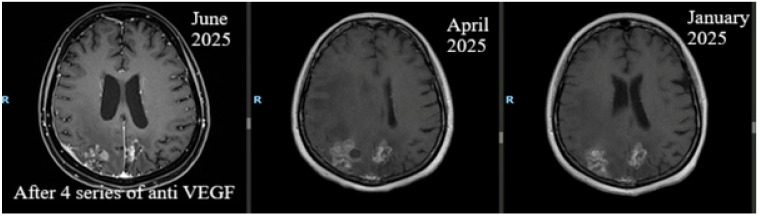
July 2025. Brain perfusion MRI after 4 cycles of treatment showing favorable overall appearance, evidenced by improved intra-axial post-therapeutic changes.

**Figure 4:**
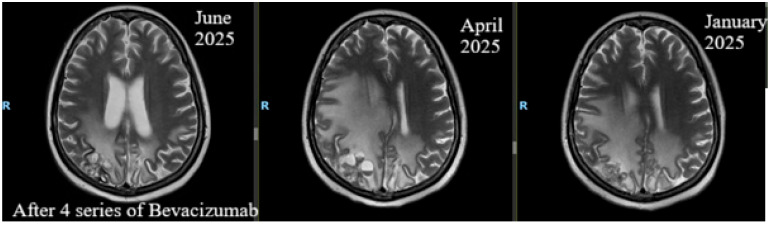
July 2025. Brain perfusion MRI after 4 cycles of treatment showing favorable overall appearance, evidenced by improved intra-axial post-therapeutic changes.

Following the change in treatment strategy, her neurological status shifted and she was able to walk without support; her Karnofsky Performance Status increased to 80–90%. In July 2025, after four cycles of treatment, brain perfusion MRI showed global improvements in intra-axial post-therapeutic changes, but pachymeningeal thickening persisted (Table 1, Figure 3 and Figure 4).

The patient continued treatment, receiving a fifth and sixth course of bevacizumab. In October 2025, brain perfusion MRI demonstrated stable bilateral parietal intra-axial changes, without new lesions and without signs of recurrence in the cerebral parenchyma. Dimensional augmentation of the right frontoparietal pachymeningeal lesion within the right frontal nodular area, adjacent to the main motor area, was suggestive of tumor recurrence. On the perfusion sequences, hyperperfusion was observed, suggestive of disease progression at this level. In this clinical and imaging context, the case was considered to be oligoprogressive disease. Brain biopsy was proposed but refused by the patient. In October 2025, PET-CT showed no metabolic activity in the visceral organs that would suggest suspicious oncological aspects. The patient refused injectable systemic oncological treatment and continued with Fulvestrant and ovarian suppression therapy.

**Table 1:** Concise timetable of events and treatments administered.

	Moment in Time	Event
1.	2018	Stage cT2N1M0, Luminal B, histopathologic result was yp T0N1(1+/4N) L0V0R0; IHC: RE95%, RP25%; HER2 = 1+; ki67 = 45%; HT. After surgery, the histopathological exam identified lymph node metastases and HER 2 status was negative.
2.	2018	Neoadjuvant chemotherapy treatment, surgery-conservative treatment, left sectorectomy and sentinel lymph node.
3.	2020	Brain CT revealed M1BRA—left and right frontal, left and right parietal, and right temporal lesions. Whole-brain adjuvant radiotherapy TD = 30 Gy/10 fractions. Systemic therapy with a CDK4/6 inhibitor plus Fulvestrant and ovarian suppression therapy.
4.	2023	February: Stereotactic radiotherapy (SRT) on left parietal brain metastases, 21 Gy/3 fractions.
5.	2024	October: brain MRI: slightly increase in right brain lesion. November: stereotactic radiotherapy (SRT) on right parietal brain metastases, 25 Gy/5 fractions.
6.	2025	May: neurologic hemiparesis clinical status of 4/5 brachial and 3/5 crural; possible walking with unilateral support; Karnofsky Performance Status (KPS) = 60–70%. May–July: 4 cycles of bevacizumab 400 mg/cycle, KPS = 80–90%. July: brain perfusion MRI showed improved intra-axial post-therapeutic changes, but pachymeningeal thickening persisted. October: PET-CT showed no metabolic activity. The patient refused injectable systemic oncological treatment and continued and continued with Fulvestrant and ovarian suppression therapy.

## 3. Discussion

The exact incidence of BRN following radiation therapy to the brain is unknown, with reported rates ranging from 5% to 50% [1,5,22]. The standard for diagnosing BRN is pathological evaluation, but due to complications associated with biopsy, it is not performed frequently. Diagnosis is usually based on imaging investigations [5].

BRN occurs months to years after initial radiation therapy. BRN involves a space-occupying necrotic lesion which causes mass effects and neurological dysfunction. The characteristic symptoms are headaches, cognitive impairment, seizures, features of raised intracranial pressure, apraxia, aphasia, and weakness.

According to the literature data, risk factors for BRN, from a histopathological point of view, include adrenal cell carcinoma, lung adenocarcinoma, especially for ALK rearrangement, and other mutations like HER2 amplification and BRAF V600+ mutation [23].

Moreover, treatment-related risk factors include the association between concurrent systemic therapy (chemotherapy, immunotherapy) and radiation, which increases the risk of developing BRN. Targeted therapies, HER2 antibody therapies like Trastuzumab Emtansine, and VEGF receptor tyrosine kinase inhibitors (TKIs) are associated with an increased risk of BRN. Reports of radionecrosis in patients receiving Trastuzumab Emtansine with brain stereotactic radiosurgery (SRS) have also been published [5,24,25,26,27]. Further, a number of radiotherapy-related risk factors exist, namely radiation dose and fraction size. A higher total dose and a larger dose per fraction significantly increase the risk of BRN. A greater total RT dose, V12, and V12 BED (biologic equivalent at one fraction) are related to the risk of developing BRN following brain RT. A larger irradiated volume is also a major predictive factor. Post-surgical adjuvant RT increases the incidence of BRN, while re-irradiation in cases with a history of prior WBRT, SRT, or SRS increases RN risk [26].

Ultimately, tumor size and location (larger tumor diameters and occipital and temporal lesions are at increased risk) are crucial determinants of RN risk [28,29].

From an anatomical–pathological point of view, BRN is characterized by damage to endothelial and glial cells, with fibrinoid necrosis and hemorrhage. Typically, necrosis is pauci-cellular and surrounded by glyotic brain tissue. Unlike BRN, tumor relapse shows high cellularity. Since BRN and tumor relapse may coexist, anatomical–pathological evaluation may not always provide a clear diagnosis [19,30,31].

Nonetheless, anatomical–pathological assessment represents the gold-standard evaluation tool. From an imaging point of view, conventional MRI is not always specific for BRN, as the radiological images obtained in BRN can be found in tumor recurrence or infection.

The distinction between pseudo-progression and BRN from true progression or stable disease is crucial for ensuring high-quality patient care [32,33]. It is a clinical challenge because both conditions appear nearly identical on standard contrast-enhanced MRI.

When conventional radiological images do not provide a clear diagnosis, the following is recommended:

- Radiomics (field of artificial intelligence that extracts large amounts of radiographic features and provides predictive models) [34]. Radiomics allows for the non-invasive differentiation between TP and BRN by extracting quantitative, high-dimensional data from MRI scans (T1-weighted post-contrast, T2, and FLAIR) that are not distinguishable by visual inspection alone. While TP is characterized by rapid, disorganized growth and increased blood volume, BRN involves structural damage, vascular disruption, and tissue coagulation.
- Magnetic Resonance Perfusion (a technique that provides information on blood volume, blood flow, and permeability) [14]. Regarding TP, this technique shows high relative cerebral blood volume (rCBV) and peak height due to neo-angiogenesis. Regarding BRN, this technique shows low rCBV because radiation-induced vascular damage reduces blood flow.

Brain perfusion MRI in Romania, particularly for neuro-oncology, primarily utilizes dynamic susceptibility contrast MRI (DSC-MRI). Specialized software is used for post-processing, with rCBV representing the primary parameter for differentiating tumor progression from radionecrosis.

DSC-MRI involves tracking a gadolinium bolus to measure the relative cerebral blood volume (rCBV), relative cerebral blood flow (rCBF), and mean transit time (MTT). Based on standard European and Romanian neuro-imaging protocols, the acquisition parameters for brain perfusion MRI are as follows: Scanner Field Strength: 1.5 T or 3 T; Sequence Type: T2*-weighted Gradient Echo Echo-Planar Imaging (GRE-EPI); Contrast Agent: Gadolinium-based Injection Protocol: Power injector used, 0.1 mmol/kg body weight, administered at a rate of 4–5 mL/s, followed by a saline flush; Echo Time (TE): 30–50 ms (lower for 3 T, higher for 1.5 T); Repetition Time (TR): 1–2 s (short TR to allow fast acquisition); Flip Angle: 60–70°; Matrix Size: 128 × 128; Field of View (FOV): 240 mm; Slice Thickness: 3–5 mm; Temporal Resolution: Fast, covering around 60–120 time points [35,36].

- Magnetic Resonance Spectroscopy (provides information on the metabolic composition of the tissue) [37,38]. Regarding TP, this technique shows high choline levels, indicating cell membrane turnover and low N-acetyl aspartate, suggestive of neuronal loss. Regarding BRN, this technique shows low levels of all metabolites but often a prominent Lipid/lactate peak, indicating cell death and anaerobic metabolism.
- Chemical Exchange Saturation Transfer MRI (a molecular imaging technique that studies the tumor microenvironment concentration and exchange of mobile proteins and peptides) [39]. MRI differentiates TP from BRN by detecting higher protein/peptide concentrations in tumors compared to necrotic tissue.
- Positron Emission Tomography (Fluorodeoxyglucose PET, amino acid PET) [40].

Amino acid PET is preferred over standard glucose PET because amino acids have better contrast against normal brain tissue. Regarding TP, this technique shows high amino acid uptake (high SUVmax) and an earlier peak uptake followed by a plateau or washout. Regarding BRN, this technique shows low or absent uptake compared to normal brain parenchyma.

Fluorodeoxyglucose PET measures glucose metabolism. Regarding TP, this technique shows high glucose consumption, while in BRN it shows low glucose consumption.

Last but not least, management options include observation of small asymptomatic lesions.

Recommendations for the management of symptomatic BRN are included in the international guidelines for the treatment of BRN published by The International Stereotactic Radiosurgery Society, ASCO-SNO-ASTRO, and other scientific societies [10,41].

Oral corticosteroids are considered the first-line treatment for symptomatic radiation necrosis at a dose of 0.5–1 mg/kg, with the role of reducing cerebral edema. Lower doses are preferred to high doses. Kotsarini et al.’s 2010 article is a systematic review detailing how dexamethasone has a significant impact on the interpretation of MRI scans, particularly for anti-angiogenic drugs, emphasizing that dexamethasone can even decrease tumor enhancement on contrast MRI, making tumors look smaller or disappear, even if the tumor itself is not shrinking [42]. Thus, dexamethasone may complicate the evaluation of the actual effectiveness of treatment. Due to their adverse effects, corticosteroids are associated, however, with significant morbidity and mortality [42,43,44].

Other classes of drugs, like Pentoxifylline (400 mg twice daily) and Vitamin E (400 IU twice daily), may be beneficial in the management of adverse radiation effects [45].

Bevacizumab (7.5 mg/kg every 3 weeks or 5 mg/kg every 2 weeks) is an effective therapy for symptomatic brain radionecrosis, reducing edema and improving neurological symptoms. Anti-angiogenic agents do not exhibit the same adverse effects of corticosteroids and therefore may be a reasonable alternative [40,46,47].

Hyperbaric Oxygen Therapy has demonstrated benefit in some cases, but it is not supported by prospective randomized studies for BRN. The evidence is limited to retrospective series and case reports, proposing it as a safe and effective treatment for BRN that demonstrates clinical and radiological improvement in most patients. Future studies are needed to assess its effectiveness and adverse effects [48,49].

Surgical resection (for refractory BRN to medical management, with the role of removing the necrotic tissue and reducing mass effects) [50] is indicated only in large lesions, hemorrhagic complications, or brain abscess [51]. Surgery thus plays a therapeutic and diagnostic role.

Laser Interstitial Thermal Therapy uses ablative hyperthermia generated by laser electromagnetic radiation to induce coagulative necrosis [52]. It is an option for unresectable cases.

Both bevacizumab and Laser Interstitial Thermal Therapy have shown favorable clinical and radiological results in the management of BRN. Bevacizumab was found to be associated with better symptom control compared to Laser Interstitial Thermal Therapy. Patient-related factors, diagnosis, and injury should be considered when choosing the ideal treatment for BRN.

BRN management options depending on the grade, according to The International Stereotactic Radiosurgery Society, are detailed in Table 2.

We reviewed the most relevant case reports and selected five studies with clinical impact published in the last 3 years. The main updates comprise BRN in nasopharyngeal cancer, squamous cell lung carcinoma, high-grade glioma, unspecified glioma, and metastatic renal carcinoma. These selected articles are synthesized and listed in Table 3.

Jlailati, A. et al., in their article published in 2025, presented the case of a 49-year-old child with nasopharyngeal cancer and BRM and highlighted the importance of early detection of BRM and follow-up through MRI and magnetic resonance spectroscopy [53].

Gan, K. et al., in their article published in 2025, presented the case of a 67-year-old man and highlighted that bevacizumab is a future therapeutic option for managing BRM in patients with squamous cell lung cancer [54]. As in the case of the patient in our study, improvement in clinical status and imaging results occurred after the administration of four cycles of bevacizumab.

Dos Santos, AAA et al., in their article published in 2025, presented the case of a 56-year-old female and showed that the accuracy of diagnosis by magnetic resonance spectroscopy and perfusion is essential to distinguish BRN from TP [55].

Ioschici, M et al., in their article published in 2024, presented the case of a 44-year-old man diagnosed with unspecified glioma and showed the importance of multimodal treatment in BRN, using bevacizumab, Hyperbaric Oxygen Therapy, and Boswellia Serrata (a tree with a resinous sap that contains several acids with anti-inflammatory properties). Boswellia Serrata could be an option in the treatment of BRN, allowing for a reduction in the doses of corticosteroids [56].

Lolli, J et al., in their article published in 2023, presented the case of a 65-year-old woman with metastatic renal cancer and BRM, and highlighted that under treatment with Cabozantinib, a reduction in BRN was observed, showing that, like bevacizumab, Cabozantinib could play a role in reducing BRN in addition to exerting systemic effects [57].

Lee SH et al. conducted a retrospective study (between September 2019 and February 2021) of electronic medical records of 45 patients diagnosed with BRN showing lesion-related neurological symptoms and subsequently treated with bevacizumab. All patients had previously undergone WBRT, fractionated local-field radiation therapy, proton beam therapy, SRS, or a combination of these modalities. The patients received bevacizumab therapy after confirming that steroid treatment had not significantly improved symptoms or MRI findings. Bevacizumab was given at a dose of 7.5 mg/kg at 3-week intervals (four-cycle schedule). The initial response of RN to bevacizumab was favorable, with improvement seen in 36 patients (80.0%) [58].

Zhuang H et al. conducted a study (between December 2016 and February 2019) on 21 patients who received stereotactic radiotherapy (SRT) and developed BRN. Twenty patients were symptomatic due to BRN. They were treated with ultra-low-dose bevacizumab at 1 mg/kg body weight (three-cycle schedule), with one infusion every three weeks. Symptoms were alleviated in 18 patients (90%) [59].

In our case, the main risk factor is re-irradiation (WBRT in 2020 followed by SRT in 2023 and 2024).

The particularities of our case are as follows:

Our patient showed long-term survival of 8 years (2018–2026) from the moment of diagnosis and 6 years (2020–2026) from the moment of diagnosis of brain metastases, with the median survival for hormone receptor-positive metastasis being around 10–18 months.

Moreover, our patient showed a favorable response to bevacizumab treatment across six cycles for BRN, with significant improvement in neurological symptoms and imaging, which shows a real benefit, even if it was off-label.

Time will tell about the benefits and overall survival of this patient.

Our patient refused brain biopsy and injectable systemic oncological treatment, which represents a diagnostic dilemma. Brain perfusion MRI from October 2025 was considered persuasive due to the dimensional augmentation of the right frontoparietal pachymeningeal lesion within the right frontal nodular area. However, it is possible that corticosteroids have an impact on the interpretation of MRI scans, highlighting that corticosteroids can even decrease tumor enhancement on contrast MRI.

Limitations of study: Although advanced imaging techniques provide valuable information, there was no histopathological verification of the final diagnosis. As brain biopsy was refused, the diagnosis remains presumptive.

## 4. Conclusions

Differentiating radiation necrosis from a recurrent tumor is crucial for appropriate treatment. Multimodal imaging improves diagnostic accuracy and plays an important role in distinguishing BRN from tumor relapse. When uncertainty exists, MRI perfusion should form part of a standard protocol, and when it is feasible, histopathology should remain the gold standard.

The case we presented is a clinically plausible mixed or evolving scenario in which radionecrosis and tumor progression may have coexisted.

Future research will play a role in improving diagnostic accuracy and therapeutic management.

## Figures and Tables

**Table 2:** BRN management depending on BRN grade.

BRN Grade	BRN Grade Definition	BRN Management
1	Grade 1 is asymptomatic RN with no prior corticosteroid use	Close surveillance with consideration of corticosteroids.
2	Grade 2 is symptomatic RN with no prior corticosteroid use	Corticosteroids are recommended.
3	Grade 3 is symptomatic RN and steroid-refractory	Bevacizumab, per the guidelines, has the most evidence, but LITT, surgery, and HBOT may also be considered.
4	Grade 4 is symptomatic RN with neurological impairment and progressive necrosis	Despite trials of non-invasive treatments, surgical resection is recommended.

**Table 3:** Comparative analytical data.

No.	Author, Year	Subject	Reference
1	Jlailati, A. et al., 2025	Radiation-induced temporal lobe necrosis in a nasopharyngeal cancer patient	[53]
2	Gan, K. et al., 2025	Brain radiation necrosis in a patient with advanced squamous cell lung carcinoma	[54]
3	Dos Santos, AAA et al., 2025	Cerebral radionecrosis in brain tumors	[55]
4	Ioschici, Met al., 2024	Use of Boswellia Serrata for cerebral radiation necrosis	[56]
5	Lolli, Jet al., 2023	Brain radionecrosis in metastatic renal carcinoma	[57]

## Data Availability

All data were collected from the patient’s medical files. As a result, all information is private and only available according to the institutional rules of “Saint Apostle Andrei” County Emergency Clinical Hospital from Galati. Before beginning any oncological treatment, the patient signed an informed consent form detailing the treatment and possible side effects. The patient gave her consent to use her medical data during the research process.
